# Observing phubbing behaviors during casual and serious conversations: consequences for conversation quality, connectedness, and appropriateness

**DOI:** 10.1186/s40359-025-02426-4

**Published:** 2025-02-27

**Authors:** Anja Stevic, Hanna Liftinger, Jörg Matthes

**Affiliations:** 1 Stanford University, Stanford, USA; 2 University of Vienna, Vienna, Austria

**Keywords:** Phubbing, Experimental study, Problematic smartphone use, Observer perspective, Social interaction, Interrupted conversation

## Abstract

The present study investigated observers’ perspectives of smartphone use during social interactions in serious and casual conversational contexts, suggesting gender differences. The results of the between-subjects 2 × 2 experimental study show that female observers perceive lower conversation quality when observing phubbing than male observers, aligning with the need-threat model’s assertion of female susceptibility to social exclusion. Moreover, observing phubbing diminishes perceived appropriateness of the interaction. Interestingly, no disparity was found in casual versus serious topics of the conversations. Societal implications are discussed.

Over the last years, research has focused on the negative effects of using smartphone during conversations [1]. Researchers refer to these phenomena as phubbing, a combination of two words, phone and snubbing [2]. Phubbing is defined as a situation in which one person starts ignoring a conversational partner and uses their phone instead of engaging in the conversation. Studies have confirmed that people find such instances of focusing on smartphones instead of partners during conversations to be rude and disruptive [3–5] and report that the quality of their social interactions and relationships suffer as a result [6–10]. In addition, phubbing negatively affects the impression that interlocutors leave [5], makes people feel socially excluded and leads to negative mood and lower well-being [6, 11, 12]. Such experiences might diminish quality of conversation, connectedness between the conversation partners, and overall perception of appropriateness to use technology during interactions. Therefore, this study aims to address key questions surrounding the impact of phubbing on interpersonal interactions. Specifically, it seeks to explore how observing phubbing affects the perceived connectedness and appropriateness of conversations between two partners. Furthermore, the study examines the role of perceived conversational quality in mediating these relationships. An additional focus is to understand how these relationships may differ depending on participants’ gender.

Understanding observers’ reactions of phubbing situations might be indicative of perceived social norms surrounding mobile etiquette [15] and point to the situational sensitivity in interpersonal conversations. Rather than examining own phubbing behavior or experiences that might be biased and inaccurately reported, we aim to test objective responses to seeing such situations during a conversation. Indeed, little is known about third-person perspectives of phubbing, with only one study showing negative outcomes based on observing photographs of smartphone use during conversation [13]. Drawing from social learning theory [27], individuals tend to model their behavior based on cues from their social environment. Therefore, witnessing phubbing behavior—where people disengage from face-to-face interactions in favor of their phones—might be an initial instance where observers notice and evaluate this behavior. Social learning theory [27] highlights that observed behaviors inform attitudes about what is normative or acceptable, even if those behaviors are evaluated negatively. In this context, observing phubbing may lead to the perception that such behavior is a common social practice, which can in turn influence attitudes and perceived norms without necessitating direct behavioral replication. Notably, Pourrazavi et al. [38] found that observational learning significantly predicts problematic smartphone use among young people. This evidence suggests that observing others engaged in phubbing may reinforce and even legitimize one’s own phubbing in social settings. Moreover, it is not clear whether observing phubbing behavior during a conversation bears similar negative consequences for both female and male observers, especially with regard to the context of the conversations, i.e., casual or serious conversational topics.

Phubbing, as a prevalent social occurrence, has been researched extensively with focus on determinants that might lead to this type of behavior, such as problematic smartphone use [14], as well as the consequences of phubbing on individuals who receive and experience this behavior from their conversation partners [6]. To integrate various mechanisms that are occurring during phubbing situations, Vanden Abeele [15] developed an overarching theoretical attention-arousal-attribution framework that explains various outcomes of the co-present smartphone use. The three sociocognitive mechanisms that occur during phubbing are: (1) expectancy violations (expectations about appropriate behavior during a conversation are disrupted), (2) ostracism (exclusion from conversation), and (3) attentional conflict (when several elements compete for individual’s attention during a conversation).

According to the theoretical framework of phubbing [15], social ostracism is one of the main mechanisms that explain harmful outcomes of being phubbed. The temporal need-threat model of ostracism is defined as a process of social exclusion and rejection [16]. Williams developed the model consisting of three consecutive stages that occur after experiencing social exclusion in terms of affective responses, delayed coping responses, and long-term consequences on mental health. There are several negative consequences which stem from being socially ignored or excluded, mainly the ones related to basic human needs, such as decreased need to belong [16, 17]. Additionally, previous research has shown that the need to belong is indeed threatened by the co-present smartphone use [6, 18]. Scholars have ascertained that phubbing became a social norm [2], implying that individuals became less prone to its effects, or even accustomed to phubbing situations. However, it is not clear how observers of phubbing interpret and react to social ostracism situations. Observers might identify or empathize with individuals that are being phubbed, especially if they deem the conversation as a more negative experience than when phubbing is absent during the conversation.

Given that phubbing is a form of multitasking, a quick glance at the smartphone during a conversation and a quick reply to a message means the attention must be divided between the two parallel actions: the real-life interaction and the online interaction [9]. This is cognitively demanding and usually leads to poorer performance in both actions [20]. If one conversation partner reaches for a smartphone, the real-life interaction has to be paused and the conversation can no longer be attentively continued. The person doing the phubbing is physically present, but cognitively in a state of “absent presence” [21]. The natural flow of the conversation is therefore interrupted. This can lead to the quality of the conversation being rated lower [6]. Expectancy violation theory [22] explains how and why norm-violating behavior could harm social interactions. Individuals possess a preconceived notion of how conversations are supposed to unfold. When non-verbal interruptions violate these anticipated social norms during conversations, individuals may experience discomfort. Behavior that deviates from what is appropriate triggers distraction, typically resulting in negative evaluation of the violator’s behavior. The theory further suggests that violating the expectations can potentially harm interpersonal interactions. In the context of phubbing, the person who is phubbing is negatively evaluated due to the act of ignoring the conversation partner [15]. Still, it is less clear whether the decreased conversation quality due to phubbing is also experienced by observers, who are not directly affected by phubbing, and whether gender and the topic of conversation plays a role in this perception.

Personal exchange and attentive listening are prerequisites for developing and maintaining interpersonal relationships [23]. Attention is expressed through the signals such as eye contact, body posture, reaction to what is said. If one interlocutor is distracted, however, these signals are disturbed and attention can no longer be signaled appropriately [9]. It has already been shown in several studies that phubbing has negative effects on relationship quality and related constructs such as closeness, affiliation, and connectedness between interlocutors [9, 19, 24]. Studies show that even the mere presence of a smartphone as a disruptive factor can be sufficient to decrease relationship quality, especially during serious, more meaningful conversations where phubbing occurs less frequently [25, 26]. 

According to social learning theory [27], people tend to acquire behavior from others through attentive observation, retention, and reproduction of observed behavior and its outcomes. The observation of others can motivate individuals to imitate the same behavior. Therefore, observing phubbing behavior in social interactions may have indirect influence on how individuals behave in the future. Based on the need-threat model [17], individuals tend to be acutely attuned to social exclusion, whether experienced directly or indirectly. The breach of social norms during interactions can result in harm to the fundamental need to belong, thereby negatively impacting individuals. The fact that phubbing has become a widespread social practice in our society has already been demonstrated [9, 28]. However, it is still unclear to what extent this behavior is deemed appropriate and socially accepted in the eyes of the observers.

Given that phubbing has a potential to disrupt conversations, in this study we are focusing on three distinct outcomes that reflect processes of perceiving phubbing interruptions. First, perceived conversation quality, defined as the observed quality of interpersonal communication that assesses aspects such as clarity, understanding, and satisfaction within conversations, tries to capture how effectively individuals communicate with one another in social interactions [33]. This is also understood as perceived social competence between conversation partners [13]. 

Second, connectedness reflects sense of relatedness or interpersonal connection within a given activity and assesses how strong the involvement between two people is [34]. Prior research on phubbing has investigated these processes as part of relationship quality between conversation partners [6]. 

Third, appropriateness refers to the degree to which behaviors or communication styles are perceived as suitable or socially acceptable within a given context. It reflects how well one’s actions align with expected norms and standards in social interactions [2]. Some studies provide evidence that norms of smartphone use in social situations have changed. For example, younger people tolerate smartphone use under certain circumstances [29, 30] and phubbing is not perceived as negative by some [9]. In contrast, there are numerous studies that demonstrate the negative effects of phubbing suggesting that phubbing is not socially accepted [2, 13]. Therefore, the current state of research does not provide clear answers as to whether phubbing has become an accepted social norm and whether or not smartphone use in conversations is judged as appropriate by observers. Moreover, there is a lack of studies that show whether the topic of conversation has an impact on the observers. One study showed that experiencing phubbing lower basic needs for connections both in serious and casual conversation contexts [18]. Thus, we ask our first research question:

## RQ1

Is the topic of conversation during observing phubbing vs. not observing phubbing behavior related to (a) poor quality of the conversation, (b) less connectedness between the conversation partners, and (c) low levels of appropriateness?

In a previous experimental study, results revealed that increased phubbing negatively influenced perceived communication quality when looking at a partner that uses smartphone [6]. Similarly, an indirect effect of phubbing has been shown via decreased relationship quality that consequently lowered evaluation of the person that was phubbing, irrespective of their gender [13]. Following the theoretical framework and previous findings, we predict the following:

## H1

Observing phubbing is related to perceived (a) poor quality of the conversation, (b) less connectedness between the conversation partners, and (c) low levels of appropriateness.

In an experimental study on observing phubbing behavior [13], conversation quality was hypothesized to mediate the relationship between phubbing and observers’ perceptions of the relationship quality between the conversation partners. The findings indicated that observers tended to evaluate those using smartphones during social interactions more negatively, particularly in terms of warmth and competence. These negative evaluations were mediated by the observers’ perceptions of the relationship quality between the individuals being observed. The study highlighted the significant role of perceived relationship quality in shaping how phubbing influenced judgments of warmth and competence. Accordingly, we hypothesize:

## H2

Perceived poor quality of conversation will mediate the association between observed phubbing and (a) connectedness as well as (b) appropriateness.

Additionally, prior research suggested potential gender differences. Generally, female participants utilize smartphones more frequently compared to males, thus females tend to engage in phubbing more frequently [2]. However, some studies argue that experiencing social exclusion affects sexes differently [31]. For instance, females tend to experience greater perceived need-threat and emotional distress and feel more hurt due to smartphone ostracism than males [18]. This sensitivity could also result in gender-based differences when observing phubbing behavior. While some studies have revealed gender differences in response to ostracism, the literature on gender distinctions in observing phubbing is less clear. One recent study found that affective and cognitive outcomes of observing phubbing was independent of observers’ gender [13]. Still, negative consequences of seeing parents’ phubbing was found to be stronger in female adolescents [32]. Due to inconsistent findings, we ask:

## RQ2

Does gender moderate the relationship between observing phubbing and (a) perceptions of conversation quality, (b) perceptions of connectedness between conversation partners, and (c) perceptions of the appropriateness of the interaction?

To answer our research questions and test our hypotheses, we relied on an experimental study and used prerecorded videos to investigate how observing phubbing behavior during a conversation between two interlocutors, in which one of the two interlocutors phubs the other, affects perceived conversation quality, connectedness and appropriateness. For comparison, we included a social interaction in which the same person listens attentively to her counterpart. In addition to observing phubbing behavior, we have also included two different topics of the conversation, based on the assumption that use of smartphones in the context of a serious conversation has more negative effects than during a casual conversation [18, 19]. The aim of our study is to explore broader negative consequences that arise from observing phubbing behaviors during social interactions by focusing on the observers’ perspective, thus expanding both theoretical and practical implications of smartphone use in society.

## Method

We conducted a 2 (phubbing vs. no phubbing) x 2 (casual vs. serious conversational topic) between-subjects randomized online experiment. Before the start of the data collection, we have obtained an IRB approval from the Department of Communication at the University of Vienna (ID: 20210527_036). To measure observed phubbing as an independent variable, we recorded twelve video stimuli, each lasting approximately 1 min and 15 sec. Each video featured two female interlocutors—a researcher and a confederate who had not participated in the survey. This choice of female conversation partners was based on the preference for female interlocutors as noted in previous research [33]. Both interlocutors followed a written script for the dialogue.

In the videos, two interlocutors were having a serious or a casual conversation during which one of the interlocutors was phubbing the other person two times for 15 sec. Participants in each of the four experimental conditions were exposed to three videos in a random order that differentiated between three conversational topics. For casual conversations, topics included a neighbor getting a new dog, a bike trip, and discovering a new restaurant. For serious conversations, topics included the corona virus, abortion, and failing an exam. Each video showed an excerpt of a conversation between two people where, in the phubbing condition, interlocutor A used a smartphone while interlocutor B was talking. In the condition with no phubbing, the same conversations were carried out and a phone was not used.

### Participants

We had a total of *N* = 263 participants in the survey experiment, equally distributed across four experimental groups, including 151 participants that identified as female (57.41%), 110 participants identified as male (41.83%) and two participants identified as diverse gender (i.e., non-binary) (0.76%). The participants were between 18 and 70 years old (*M* = 36.83). Almost half (48.67%) had high educational level (i.e., completed a university or to have graduated). All participants were active smartphone users: 11.4% indicated using smartphone for one hour a day, 29.3% indicated using smartphone for 1–2 h per day, 26.2% used smartphone for 2–3 h a day, 14.8% used smartphone for 3–4 h daily, 10.6% used smartphone for 4–5 h a day, and 7.6% indicated using smartphone for more than 5 h a day.

Manipulation check was successful for both conditions. All participants reported seeing smartphone use in the videos in the phubbing condition and correctly assessed whether topics of conversation were casual or serious, t-test confirmed the significant difference between the two groups, *t*(242.27) = −28.11, *p* <.001.

### Measures

After exposure to videos, we measured *perceived conversation quality* with seven items from the Chotpitayasunondh and Douglas study [6] that used the quality of communication scale [33]. We asked participants to rate the observed conversation on a scale with attributes such as, smooth – difficult, open – distant (range 1–7; α = 0.86, *M* = 2.7, *SD* = 1.02). Higher scores indicated poorer conversation quality. We assessed perceived *connectedness* between the two interlocutors with six items from the connectedness subscale of the intrinsic motivation inventory [34], e.g., “Both conversation partners feel connected to each other” (α = 0.89, *M* = 4.89, *SD* = 1.31). *Appropriateness* was measured with two items adapted to the perceived appropriateness of the interlocutor A that was phubbing in the video, i.e., “Interlocutor A behaved appropriately during the conversation” (range 1–7, *r* = .88, *p* <.001, *M* = 4.4, *SD* = 2.2).

### Data Analysis

We conducted two moderated mediation analysis in SPSS PROCESS Model 8 [35] with two dependent variables, connectedness and appropriateness. Observed phubbing and topic of conversation were used as independent variables. Conversation quality was used as a mediator and gender variable was used as a moderator as well as the conversation topic. We controlled for participants’ smartphone use, age, and educational level.

## Results

For RQ1, we found no significant outcomes of the topic of conversation, thus there were no differences regarding casual or serious conversation. Instead the findings differed only between the groups that were exposed to phubbing or no phubbing behaviors and the conversational topic did not matter for perceived conversation quality, connectedness, and appropriateness.

For H1, the results revealed that observing phubbing behavior did not relate to perception of poor conversation quality (*b* = 0.29, *SE* = 0.18, *p* = .108). Observed phubbing was not directly related to perceived connectedness (*b* = − 0.37, *SE* = 0.19, *p* = .052), however, it was directly related to lower appropriateness of the interlocutor who was phubbing (*b* = −3.29, *SE* = 0.24, *p* < .001).

For H2, perceived poor conversation quality was related to less perceived connectedness between the two conversation partners (*b* = − 0.80, *SE* = 0.07, *p* < .001) as well as to lower perception of appropriateness of the interlocutor who was phubbing (*b* = − 0.31, *SE* = 0.08, *p* = .002), showing no mediation effect.

**Fig. 1:**
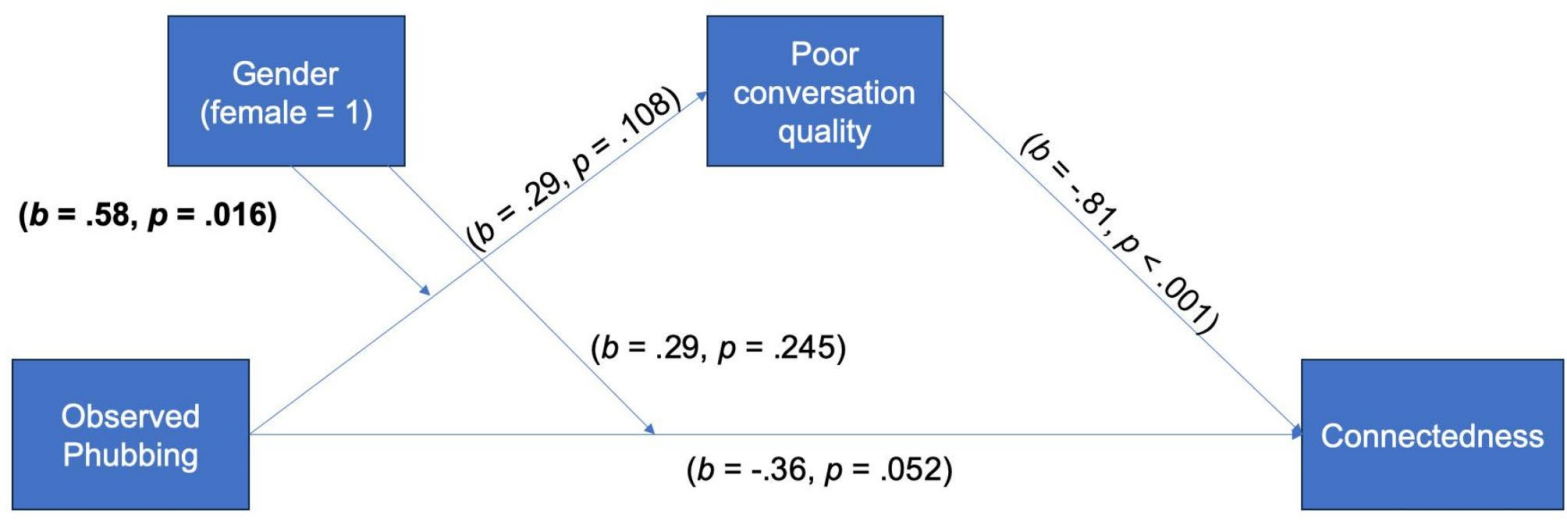
Results of the moderated mediation model with connectedness as dependent variable

**Fig. 2:**
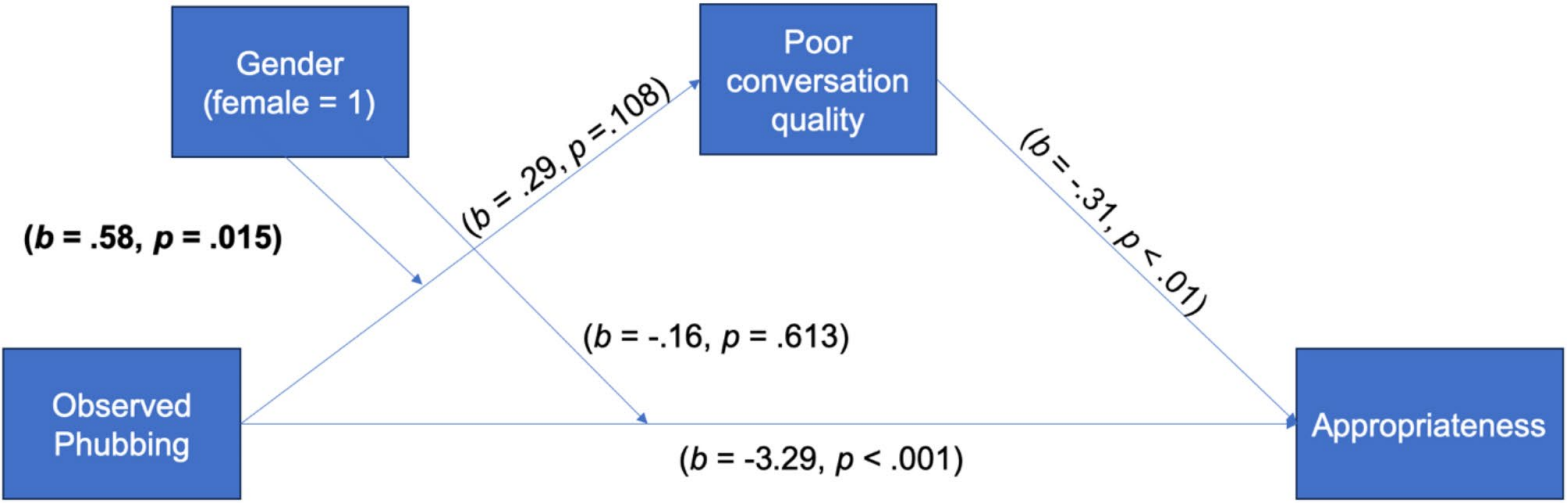
Results of the moderated mediation model with appropriateness as dependent variable

For RQ2, gender was found to moderate the relationship between observed phubbing and poor conversation quality (*b* = 0.58, *SE* = 0.24, *p* = .015). When observing phubbing behavior, females perceived lower conversation quality between the conversation partners in comparison to males (*b* = 0.87, *SE* = 0.15, *LLCI/ULCI =* 0.56/1.17). The overview of key results is presented in Figs. 1 and 2.

### Additional analyses

**Fig. 3:**
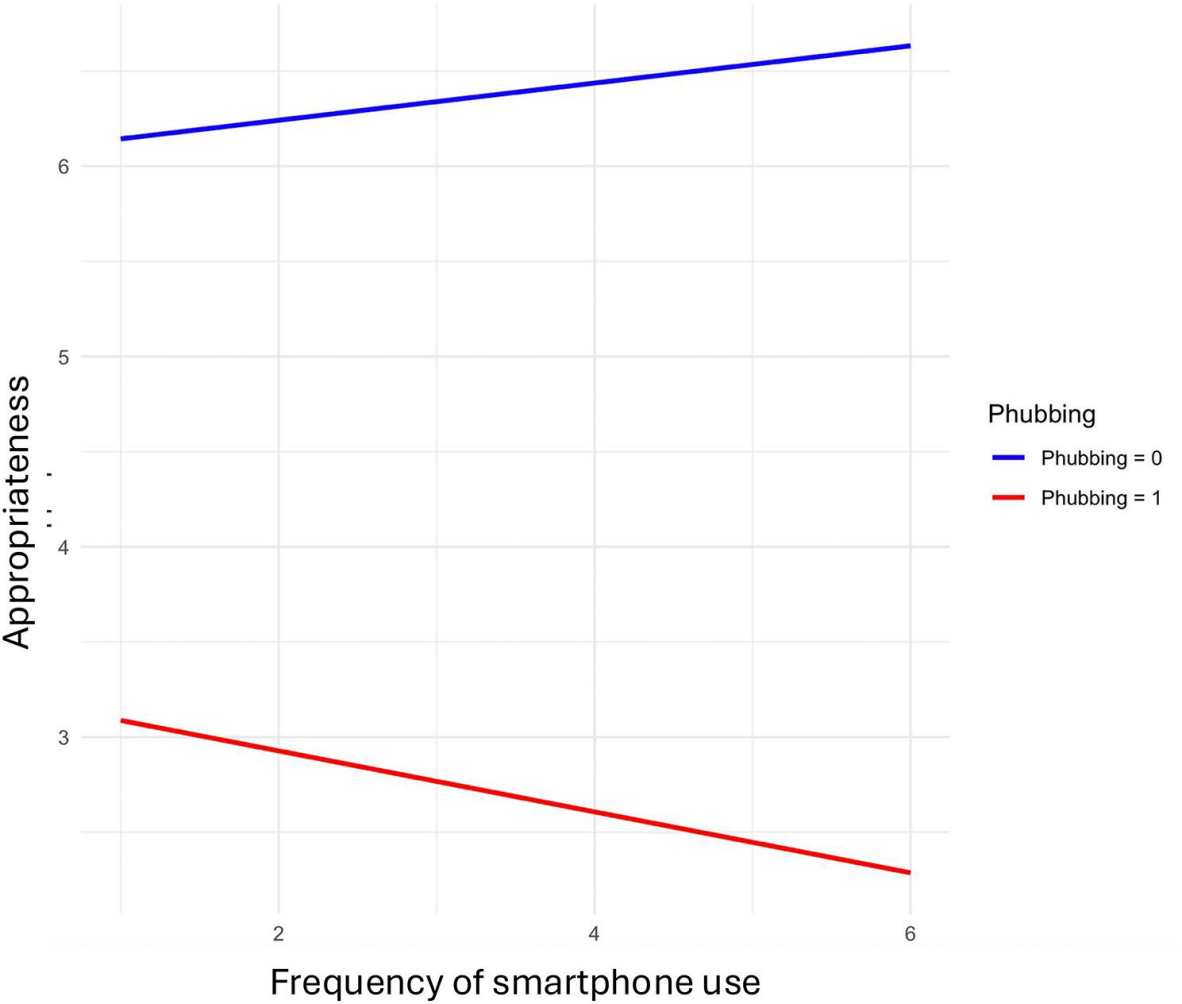
The interaction between phubbing and smartphone use predicting appropriateness

Prior research has emphasized that differences in smartphone use might influence how phubbing is perceived and evaluated [6, 14, 39]. Given that the participants reported varying amounts of their own frequency of smartphone use, we have conducted additional analyses to account for potential differences among low and high groups of smartphone users. First, we tested whether there are differences in the frequency of smartphone use among genders. The one-way ANOVA did not reveal a significant effect, *F*(2, 260) = 2.224, *p* = .11. Therefore, we did not find differences in smartphone use among genders in our data. Second, we tested smartphone use as a moderator (see Fig. 3). The interaction between phubbing and smartphone use showed a significant negative relationship predicting appropriateness, *b* = –0.25, *SE* = 0.11, *p* = .022 (*LLCI/ULCI = *− 48/–0.04). The interaction effect suggests that the influence of smartphone use on appropriateness is dependent on whether phubbing is observed or not. In situations where phubbing is present, observers’ higher smartphone use could be linked with lower appropriateness values, possibly indicating a negative outcome when both phubbing behavior and individual’s high smartphone use are present. This result indicates that observers may judge the situation as inappropriate or socially undesirable when both phubbing and high freqency of individual smartphone use are involved.

## Discussion

In this between-subjects experimental study, we have exposed participants to several prerecorded videos of conversations, to mimic an externally valid observation of a social interaction that could occur, for instance, in a restaurant or other public or semi-public setting. The aim of our study was to understand how observers of phubbing behavior evaluate a conversation between two interlocutors, while one is phubbing the other, depending on their gender and the topic of the conversation. Thus, the present study aims to advance our understanding of observing phubbing situations in two key ways: gender-related differences and the context of the conversational topic. First, we found no relationship between observed phubbing and perceived conversation quality, however, our results demonstrated that only female participants perceived lower conversation quality when observing conversation partners in which one interlocutor is phubbing the other person. This finding is in line with the theoretical assumptions of the need-threat model, which suggests that females are more susceptible to social exclusion than males [31]. Additionally, we extend previous findings, showing that not only experiencing phubbing from others hurts female conversation partners more than male [18], but also seeing these situations in other social interactions decreases perceived conversation quality, connectedness, and appropriateness between observed two interlocutors. One potential reason for female participants experiencing this negative outcome could be in the way they engage in more frequent smartphone use for relational maintenance and connection with others. Thus, observing the same in others could bring about similar feelings of empathy and understanding of exclusion. Additionally, the two interlocutors in the videos were both female, therefore, the female participants could have related more to them than male participants. For observers, studies have reported that conversations with female partners are preferred and have higher conversation quality [33], thus our findings show significance when females observed lower quality due to the presence of the smartphone. Future studies should account for different gender representations in the videos to test whether the findings hold when, e.g., cross-gender interlocutors have a conversation during which one is phubbing. Since our study cannot test gender as a factor according to having only one gender in the stimuli, we can only suggest gender differences among observers that are participants in the study. Future research should more systematically test gender differences including gender as a factor in stimuli design and as participant’s characteristic.

Second, we found no differences in the outcomes depending on the casual or serious topic of conversations, as shown in previous experimental study on phubbing during meaningful or casual conversations [25]. This null finding suggests that the content of conversation does not really matter when phubbing occurs. Similar results were confirmed in a study on valuing interaction [39], where decrease in phubbing behavior was not found if conversations were deemed more relevant. Another explanation could be due to the content of the topics recorded in the videos. Based on our definition of casual and serious topics, we focused on positive topics for casual conversations and on negative topics for serious conversations. Although participants could clearly differentiate between the two topics, as confirmed in the manipulation check, the relevance of the content might differ among observers. It is possible that many participants could not relate to the conversation if the topic was not personally relevant to them. To address this limitation, we propose that future studies directly assess participants’ perceived relevance of conversation topics and ensure that presented scenarios align with participants’ personal definitions of casual versus serious conversations. For example, personalized or contextually adaptive scenarios could provide deeper insights into how topic relevance interacts with phubbing’s social and psychological effects.

Furthermore, the lack of differences in perceptions based on the seriousness of conversation topics suggests that the impact of phubbing may be pervasive across various social interactions, regardless of content. This underscores the need for interventions and education aimed at promoting mindful and respectful smartphone use during interpersonal communication, regardless of the conversational context.

Our findings offer valuable insights in the context of expectancy violation theory [22, 29], which suggests that individuals hold expectations about others’ behavior in social interactions, and deviations from these expectations can lead to negative perceptions and, subsequently, influence attitudes or norms surrounding the social interaction. Vanden Abeele et al. [41] argue that if phubbing, as a disruption, induces an expectancy violation experience in interactions with strangers—similar to what was demonstrated in early experiments on interpersonal expectancy violations—it would further reinforce the notion that phubbing breaches general social norms, rather than norms specific to work or social relationships. This suggests that while the impact of observing unaffiliated others may be less pronounced, it remains significant in terms of norms. Although we did not measure participants’ social norms before they watched the videos or tracked behavioral changes after viewing them, recognizing negative impacts on the conversation represents an initial step in observational learning that could shape future attitudes. Future research could assess observers’ expectations of social norms prior to showing them videos, testing whether holding different norms affects their perceptions of conversational quality. This approach could provide further support for non-verbal expectancy violation theory.

Additionally, this study did not find evidence for the hypothesized mediation path between observed phubbing, conversation quality, and the outcomes in terms of appropriateness and connectedness among third-party observers. One possible explanation for this null finding is that the observers had no real-life connection to either conversation partner. Prior research has shown that individuals in varying social contexts—such as within families, among friends, between romantic partners, or in professional relationships—perceive phubbing and its impacts on conversation and relationship quality differently [37]. We can thus anticipate variations in observers’ perceptions as well; for example, observing a mother phubbing grandparents at a family gathering may have different implications than witnessing a friend phubbing another friend that can be more acceptable. These situations suggest that perceptions of phubbing may vary depending on the relationship strength and affiliation between the individuals involved, as well as based on observers’ own social contexts and firsthand experiences.

The societal implications of these findings shed light on how co-present smartphone use, particularly in terms of distraction and interruption of face-to-face conversation, impacts observations of social ostracism [36]. As established in the need-threat model [17], individuals are susceptible to social exclusions even when they are experienced indirectly [13]. Extending previous findings to third-person perspectives, strengthens our understanding of negative societal impact that phubbing has during social interactions not only on the phubbees but also on the observers of those interactions, particularly female. Therefore, understanding differences in the perception of phubbing can inform interventions aimed at mitigating its negative effects, particularly in contexts where female conversation partners may feel more vulnerable to social exclusion. As smartphones increasingly mediate social interactions, awareness of how observing phubbing in others affects perceived connectedness and conversation quality between two interlocutors can inform etiquette guidelines and social norms surrounding smartphone use.

## Limitations

Our experimental study has several limitations, notably the absence of assessing behavioral changes resulting from observations of phubbing others. While the study systematically examined participants’ perceptions of conversation quality, connectedness, and appropriateness of other’s social interactions, it did not measure potential alterations in actual behavior following exposure to phubbing scenarios. Future research could address this gap by incorporating behavioral metrics and testing social norms of phubbing, to provide a more comprehensive understanding of how observing phubbing influences perceived norms and potential changes of own smartphone use during social interactions.

Moreover, through our research study design it is not clear whether having affiliation or no affiliation to the observed conversation partners impacted the perceptions of connectedness and quality of social interaction, since participants were unrelated to the persons in the prerecorded videos. Meta-analytic evidence suggests that closeness and affiliation to others, increases negative outcomes of phubbing [40], thus testing this component is relevant for future studies.

Additionally, we focused only on the short-term consequences of phubbing. Generally, little attention has been paid to the long-term consequences. The only exception is a panel study by Halpern and Katz [7], in which long-term effects of phubbing between romantic couples were examined. Due to the methodologically complex implementation, we could not investigate the long-term causal effects of phubbing in this experiment. However, in order to capture the phenomenon of phubbing comprehensively, future studies could investigate how phubbing affects observers when they are exposed to it over a longer period of time, e.g., through frequent real-life exposures or via media portrayals of phone use in conversations.

## Conclusion

This experimental study used prerecorded videos to investigate how observing phubbing behaviors during a social interaction between two interlocutors affects perceived conversation quality, connectedness and appropriateness. We have also tested whether gender and serious or casual conversation topics make a difference. Taken together, our study shows no differences regarding the contextual outcomes of phubbing behavior during casual or serious conversation, demonstrating that the negative outcomes regarding phubbing behaviors are universal no matter the content of a conversation. However, we also show that female observers perceive phubbing more as having negative consequences than male observers, emphasizing gender-related differences regarding situations of social exclusion.

## Data Availability

Data is available upon request.
